# Lipids regulate epidermal growth factor receptor activation by its ligands

**DOI:** 10.1042/BST20253090

**Published:** 2026-01-22

**Authors:** James M. Hutchison, Mark A. Lemmon

**Affiliations:** 1 Department of Pharmacology, Yale University School of Medicine, New Haven, CT, 06520, U.S.A.; 2 Yale Cancer Biology Institute, Yale University West Campus, West Haven, CT, 06516, U.S.A.

**Keywords:** cancer, epidermal growth factor receptor, lipid metabolism, lipids, receptor tyrosine kinases, transmembrane proteins

## Abstract

The epidermal growth factor receptor (EGFR) is a receptor tyrosine kinase that has garnered extensive interest since its discovery as an oncogene product in the 1980s. We now understand that the binding of soluble growth factors to EGFR activates it by facilitating receptor-mediated EGFR dimerization. However, how the extracellular ligand-binding and intracellular tyrosine kinase domains communicate across the bilayer remains unclear. This lack of understanding likely originates from a ‘divide and conquer’ approach that has provided a detailed understanding of the respective domains in isolation but only limited knowledge of how they are co-ordinated during signaling. Attempts to study full-length EGFR in detergents or membrane environments that lack possible key lipid cofactors leave a critical component of intact receptor signaling understudied. Indeed, multiple classes of lipids, such as gangliosides and PtdIns(4,5)*P*_2_, have long been known to influence EGFR signaling in cells, and a lack of their inclusion in *in vitro* studies has hindered mechanistic understanding of the intact receptor. This review highlights recent studies of how lipids regulate EGFR activity, with special attention paid to potentially actionable co-dependent lipid metabolism in glioblastoma multiforme and promising new methods for studying membrane protein–bilayer interactions.

## Introduction

Lipids are ubiquitous amphipathic molecules that spontaneously form bilayers impermeable to most hydrophilic molecules and create a crucial barrier between the cell interior and its external environment. *In vivo*, lipid bilayers are chemically complex environments composed of lipids, proteins, and carbohydrates that all regulate membrane protein (MP) function by defining their biochemical environment, spatial trafficking, and local proteome [1,2]. Human cells contain hundreds of chemically unique lipid species through the combinatorial assembly of various hydrophilic headgroups and hydrophobic fatty acids with varied lengths and degrees of saturation [3,4]. Bacteria, plants, and vertebrates all dedicate significant energy to metabolizing and regulating their many chemically distinct lipids [5-8]. Shouldn’t we therefore expect lipids to modulate or regulate proteins that evolved alongside changes in bilayer composition? Indeed, there is a growing appreciation that lipids directly regulate polytopic MPs that function through conformational changes within the bilayer. Examples include G protein-coupled receptors [9,10] and ion channels [11-13]. By contrast, relatively little is known about the importance of lipids for the function of receptor tyrosine kinases (RTK), bitopic MPs that have only a single membrane span and for which gross structural conformational changes and regulated enzymatic activity occur outside the bilayer itself [14]. This review highlights the importance of lipids for RTK signaling by focusing on the epidermal growth factor receptor (EGFR) and connections between its regulation in cancer and lipid metabolism—while summarizing new technologies that may enable the study of EGFR and related RTKs in their native lipid environment. Computational studies have greatly advanced our thinking about the structure and dynamics of membranes [15] and have been extended to lipid interactions with, and influence on, EGFR [16-18], but we mostly restrict ourselves in this short review to complementary experimental studies. Since this is a brief and lipid-focused review, readers are directed elsewhere for detailed information on EGFR signaling more generally [14,19-22].

**Figure 1:**
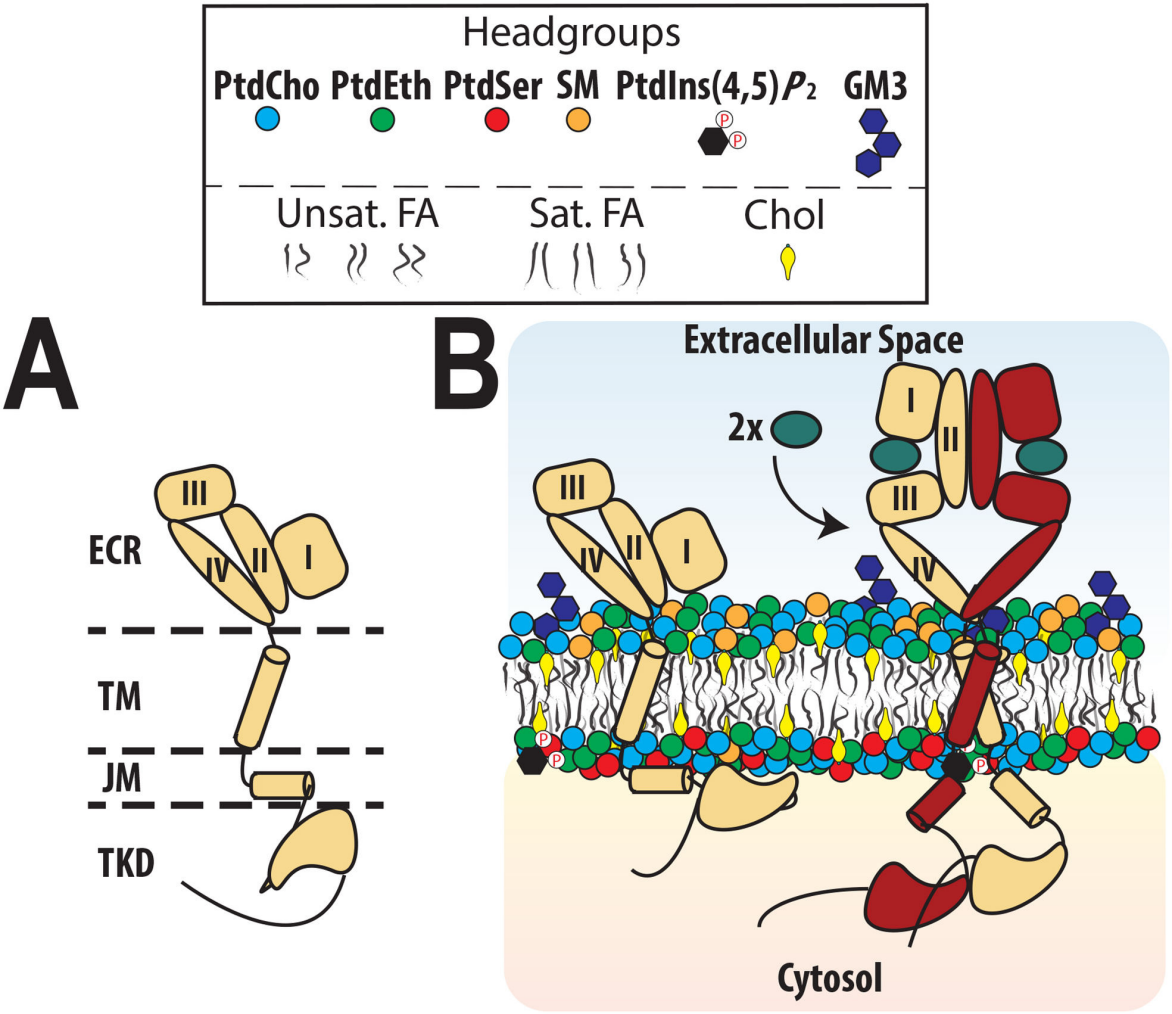
EGFR domain organization and activation. (**A**) EGFR consists of an extracellular region (ECR) with four domains (**I-IV**) connected by a single transmembrane (TM) region to a cytosolic juxtamembrane (JM) region and an intracellular tyrosine kinase domain (TKD). (**B**) The addition of an activating ligand (such as EGF) results in EGFR-mediated ECR dimerization and activation of the TKD by formation of an asymmetric TKD dimer capable of autophosphorylating the unstructured C-tail *in trans*. Lipid abbreviations in the legend are: PtdCho, phosphatidylcholine; PtdEth, phosphatidylethanolamine; PtdSer, phosphatidylserine; SM, sphingomyelin; PtdIns(4,5)*P*_2_, phosphatidylinositol 4,5-bisphosphate; Chol, cholesterol; FA, fatty acid.

EGFR is the founding member of the ErbB RTK family and initiates growth factor-induced signaling across the plasma membrane to promote diverse responses from differentiation to cell division and metabolic signaling, making it an effective therapeutic target in multiple cancers [23-25]. EGFR contains a ligand-binding extracellular region (ECR) that is coupled by a single transmembrane (TM) region to an intracellular tyrosine kinase domain (TKD) and a large, disordered C-terminal tail (C-tail) through small juxtamembrane (JM) regions (**Figure 1A**). The seven known activating ligands of EGFR promote distinct cellular outcomes depending on biological context [22,26,27] by driving dimerization of the ECR that in turn promotes activation of the TKD by bringing it into an asymmetric dimer aided by JM region interactions (**Figure 1B**) [28-31]. A key gap in our understanding of EGFR activation is an appreciation of whether (or how) extracellular conformational changes are allosterically communicated to those on the other side of the plasma membrane in a stereospecific manner. Membrane lipids are clearly potential regulators of this important aspect of ligand-dependent signal transmission, but we currently lack mechanistic and quantitative details on how they might achieve this. Indeed, structural and functional studies of EGFR have typically adopted a ‘divide and conquer’ approach, delivering a detailed understanding of how the isolated EGFR domains function but little information on how they communicate with one another during signaling [30-34]. *In vitro* studies of full-length EGFR have also failed to explain receptor properties observed *in vivo*, likely because of the technical necessity to replace the native bilayer with membrane mimetics during purification. Replacing the native membrane environment in this way has created a ‘membrane blind spot’ that may hide the answers to some of EGFR’s most pertinent questions, such as, why is EGFR’s reported kinase activity so low compared with other RTKs? Why has a complete, intact RTK never been observed at atomic resolution?

### The lipid bilayer stabilizes EGFR’s kinase activity

Lipid bilayers are composed of two phospholipid leaflets; the fatty acid acyl chains form a hydrophobic core and the hydrophilic (zwitterionic, anionic, or cationic) headgroups face the aqueous environment, providing a highly charged interface for protein–lipid and counterion interactions [35]. MPs evolved within this uniquely heterogeneous and structured biological ‘solvent,’ making the lipid environment a critical partner in MP function—just as water and counterions are crucial for soluble protein function. During the *in vitro* MP extraction and purification required for studies of MPs, detergents are invariably used to replace the native bilayer environment. At best, this replacement limits our understanding of protein–lipid interactions, and at worst, it induces artifacts by destabilizing MPs [36]. Modern MP studies try to limit destabilization of folded proteins by using detergents with non-ionic headgroups such as n-dodecyl-β-maltoside that are generally less denaturing than their ionic counterparts (such as dodecylphosphocholine) [37]. Although non-ionic detergents have long been used to purify EGFR that retains some elements of function, Mi et al. demonstrated that EGFR kinase activity is lost over ~10 hours when purified in non-ionic detergents [38]. Reconstituting into bilayer-mimicking membrane scaffold protein nanodiscs based on egg phosphatidylcholine (PtdCho) mitigated this reduction in kinase activity. Thus, the presence of a lipid bilayer appears to be crucial for preserving the kinase activity of intact EGFR. Moreover, the absence of lipid cofactors, such as anionic lipids, may explain EGFR’s reputation as an anemic kinase—with a reported k_cat_ value that is ~10–20 fold lower than those reported for autophosphorylated TKDs from other RTKs such as anaplastic lymphoma kinase, tropomyosin receptor kinase A/B (TrkA/B), and the insulin receptor [39-42].

### The intracellular juxtamembrane region is a key regulatory element that interacts with anionic lipids

As zwitterionic PtdCho and phosphatidylethanolamine (PtdEth) lipids are the major bilayer building blocks of mammalian membranes, most *in vitro* studies of EGFR have been carried out in model lipid bilayers containing only these lipids. While PtdCho/PtdEth lipid models provide an appropriate lipid ‘solvent’ in a general physical sense, they lack lipid cofactors that have been reported to regulate EGFR. These reported lipid cofactors, e.g. anionic lipids and gangliosides, are asymmetrically distributed both between the two leaflets of the plasma membrane (PM) and laterally along the PM [43-45]. The cytoplasmic leaflet of the PM contains most of this membrane’s anionic lipids, including phosphatidylserine (PtdSer), phosphatidylinositol (PtdIns), and the phosphorylated PtdIns derivatives (PtdIns*P*_n_s)—and these anionic lipids play essential roles in EGFR signaling [46]. Many RTK signaling pathways, including those of EGFR, use PtdIns*P*_n_s as secondary messengers during signal propagation [14]. However, additional structural roles that involve direct interactions of anionic lipids with basic regions in the EGFR cytosolic domains remain poorly defined. Both the TKD and the JM region (residues 669–706: using UniProt numbering) of EGFR contain basic residues that are thought to interact with anionic lipids within the PM. Mutations in the JM region diminish EGFR autophosphorylation (as a measure of activity) by ~30–50%, and deleting the JM lowers kinase activity by ~95% [29,30,47,48]. The JM region is proposed to aid in orienting the TKDs of intact EGFR to form the active asymmetric dimer by balancing anionic lipid interactions with the JM-mediated interactions [29,30,47]. Furthermore, electrostatic interactions involving the JM region are subject to negative feedback through phosphorylation at T678 (by protein kinase C [PKC]) and T693 (by MAP kinases), which diminish EGFR activation by interfering with JM–anionic lipid interactions [29,49-53]. The conservation of JM basic residues between EGFR and other ErbB family members with active kinases (ErbB1, 2, and 4) also suggests that anionic lipid interactions are generally important for ErbB receptor function [29,54].

Given the importance of the EGFR JM regions, numerous groups have sought to understand their interactions with lipids biophysically. Molecular dynamics simulations support the role of anionic lipids in regulating JM-mediated interactions by finding that dimers of a helix formed by the N-terminal part of the JM region appear less stable when simulated in bilayers containing only zwitterionic PtdCho compared with a PtdCho/PtdSer mixture [47]. Numerous *in vitro* studies demonstrate that PM-abundant PtdSer (~20 mol%) and scarce phosphatidylinositol 4,5-bisphosphate [PtdIns(4,5)*P*_2_] (~2% of glycerophospholipids) both interact with basic residues in the intracellular JM region [55-58]. Isolated JM-derived peptides preferentially interact with artificial bilayers containing PtdIns(4,5)*P*_2_ over PtdSer due to differences in their net charges of nearly -4 and -1, respectively [56,57,59]. This preference for PtdIns(4,5)*P*_2_ helps explain why local PtdIns(4,5)*P*_2_ levels correlate with EGFR clustering before and during activation in cells, despite the higher concentration of PtdSer in the bilayer [55,56,60]. EGFR is thought to be recruited to PtdIns(4,5)*P*_2_ clusters that form independently through interactions with biologically relevant divalent ions [61,62]. Although interactions of the JM region with anionic lipids are well-founded biophysically and supported by cellular studies, we have yet to understand their relative importance to overall EGFR activity. There is a need for quantitative studies of full-length wildtype EGFR activity in the context of varied anionic lipid environments to conclusively demonstrate and better explore the importance of JM–lipid interactions for EGFR activity and clustering.

### GM3 inhibits EGFR, but the mechanism is unclear

Just as the EGFR intracellular region (ICR) interacts with anionic lipids in the cytoplasmic leaflet of the PM, so is EGFR’s ECR thought to interact directly with gangliosides enriched in the extracellular leaflet [45,63]. Gangliosides are broadly distributed glycosphingolipids with oligosaccharide headgroups that contain negatively charged sialic acids. Gangliosides are consistently found to alter growth factor signaling, but findings are inconsistent in whether they report up- or down-regulation of signaling for different RTKs [64]. For example, GM1 has been reported to inhibit platelet-derived growth factor receptor signaling in 3T3 fibroblasts and SH-SY5Y neuroblastoma cells but to increase TrkA signaling in PC12 cells [65-67]. One of the simplest gangliosides, GM3, has long been known to inhibit EGFR autophosphorylation when exogenously added to neuroblastoma and A431 cells. Accordingly, pharmacological reduction of GM3 levels increases EGFR autophosphorylation [68-70]. GM3 inhibits EGFR through direct interactions with the receptor that do not affect EGF binding [71]. It is unclear whether the ECR–GM3 interaction is driven by electrostatic interactions between the membrane-proximal ECR lysine K642 and the N-acetylneuraminic acid of GM3 as proposed [72,73]—similar to GM3 interaction with a JM lysine in the insulin receptor [74]—or by carbohydrate–carbohydrate interactions between EGFR N-glycans and GM3 oligosaccharides [75,76] (or both).

### Raft-like membrane environments can influence EGFR trafficking and activation

Beyond leaflet asymmetry, bilayers also contain lateral heterogeneity that may influence EGFR signaling through direct interaction of lipids with the receptor and by altering receptor trafficking. Lateral bilayer heterogeneity is often attributed to lipid–lipid phase separation, a complex topic that is covered only very briefly here. Lipid bilayers are capable of forming several distinct phases, but the liquid-ordered (Lo) and liquid-disordered (Ld) appear most relevant [77]. As the name suggests, Lo domains have more ordered lipid packing, reducing lateral diffusion and increasing bilayer thickness compared with the Ld phase. Important for this review, Lo domains are enriched in saturated glycerophospholipids, sphingolipids, and cholesterol, whereas Ld domains are enriched in unsaturated phospholipids. Lo–Ld phase separation arises from balancing minuscule attractive and repulsive forces between individual lipids that, in aggregate, result in macroscopic bilayer behaviors [78,79]. Macroscopic Lo–Ld phase separation has long been noted in artificial bilayers [80,81], and a related phenomenon can be observed in giant plasma membrane vesicles (GPMVs) when cooled [82,83]; however, phase separation has not been directly observed *in vivo* outside of yeast vacuoles [84-86]. Biological membrane domains thought to resemble Lo or Ld phases are known as lipid ‘rafts’ and bulk membrane, respectively. The differing physicochemical properties of bulk and raft-like domains, along with differences in their proteome, allow for differential regulation of associated MPs.

**Figure 2:**
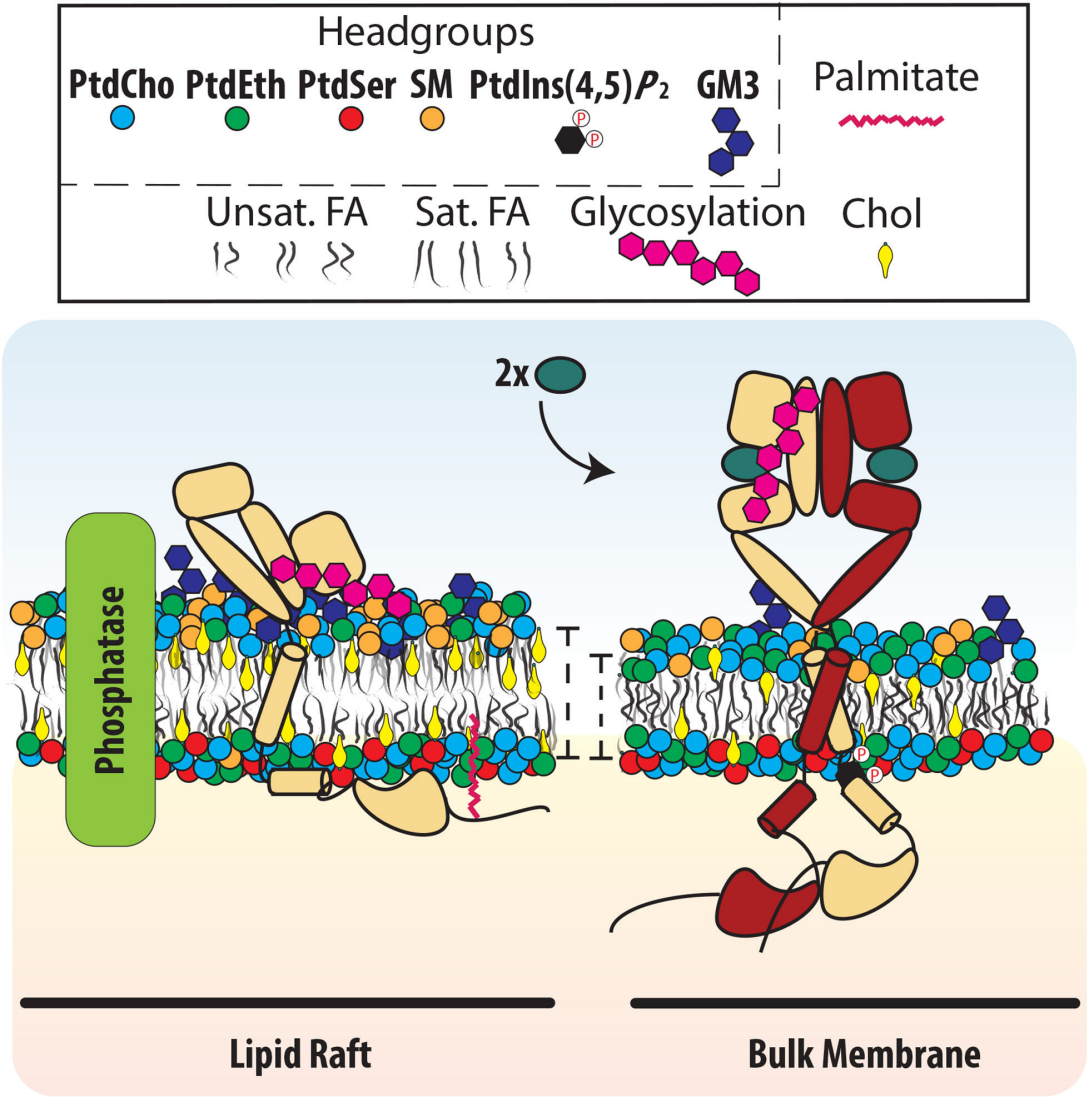
EGFR’s activation is influenced by the local lipid environment. Inactive EGFR is associated with cholesterol- and sphingolipid-rich lipid-raft silencing environments. In contrast, ligand-activated receptors tend to be associated with the bulk membrane, where this inhibition is relieved [90-92]. Cholesterol depletion disrupts raft-like domains and increases EGFR autophosphorylation by altering EGFR oligomeric states, increasing EGF binding, and retaining the receptor at the cell surface [90,93-97]. Lipid abbreviations in the legend are: PtdCho, phosphatidylcholine; PtdEth, phosphatidylethanolamine; PtdSer, phosphatidylserine; SM, sphingomyelin; PtdIns(4,5)*P*_2_, phosphatidylinositol 4,5-bisphosphate; Chol, cholesterol; FA, fatty acid.

Different MPs tend to show preferential partitioning into, or (conversely) exclusion from, raft domains of cooled biological membranes. For example, the T cell receptor tends to associate with Lo domains, supporting the idea that lipid rafts may function as signaling platforms [87-89]. By contrast, for EGFR, it has been reported that a portion of the inactive form of the receptor associates with raft-like domains, although this varies across cell lines [90-92] (**Figure 2**). Raft-like domains could act generally as ‘silencing’ platforms for EGFR signaling. Indeed, many factors known to down-regulate EGFR signaling, such as protein tyrosine phosphatases, saturated glycerophospholipids, gangliosides, sphingomyelin (SM), and cholesterol, are also enriched within raft-like domains [77,98] (**Figure 2**). The underpinnings of EGFR-raft affinity remain unclear. Several factors have been shown to increase MP raft domain partitioning, including increased TM domain length, decreased TM surface area, and palmitoylation, the post-translational addition of a palmitate to a cysteine [87,99]. EGFR has been shown to be palmitoylated by the palmitoyl transferases DHHC20 and DHHC13 at multiple cysteines in its C-tail, possibly helping to explain the association between EGFR and raft-like domains. Data on the impact of palmitoylation on EGFR activity are conflicting, but there is agreement that palmitoylation plays an important role in EGFR trafficking [100-103]. Palmitoylation is commonly found to be a regulator of MP trafficking, and altering the distribution of EGFR at the PM would certainly affect its signaling [104].

If modifying EGFR’s association with raft-like domains can influence signaling by altering receptor trafficking, then changing the abundance of lipids involved in raft formation, e.g. cholesterol, SM, and saturated glycerophospholipids, should similarly influence EGFR activity. Fast-dividing cancer cells require high cholesterol levels for membrane biogenesis and are routinely shown to up-regulate cholesterol biosynthesis and uptake [105]. Increased cellular cholesterol tends to dampen EGFR signaling, whereas cholesterol depletion tends to increase EGFR autophosphorylation—apparently by altering EGFR oligomeric states, increasing EGF binding, and retaining receptors at the cell surface [90,93-97]. Like PtdCho and PtdEth, cholesterol likely does not influence EGFR directly but by altering the distribution of the receptor in cell membranes. Spatial regulation of EGFR by cholesterol could occur at the organellar level, as with SM d18:1/n16:0 discussed below, or within the PM through cholesterol-dependent raft-like domains. Lowering membrane cholesterol is hypothesized to reduce raft microdomains, allowing EGFR to diffuse away from high concentrations of raft-associated factors that silence signaling. Coskun et al. demonstrated how the inhibitory effect of GM3 only occurs in vesicles with lipid compositions that allow Lo–Ld phase separation [72]. Moreover, high cholesterol concentrations are frequently associated with EGFR resistance to tyrosine kinase inhibitors (TKIs) in lung cancer cell lines. Lowering cellular cholesterol in TKI-resistant cell lines with methyl-β-cyclodextrin or statin treatment increases EGFR response to ligand while also resensitizing cells to TKIs [106,107]. Furthermore, a retrospective cohort study found that patients taking statins during TKI therapy often have better outcomes than those taking TKIs alone [108]. These studies strongly imply that the degree of EGFR–lipid raft association alters receptor signaling and suggest that targeting cancer-related aberrant lipid metabolism might be beneficial.

### EGFR signaling influences, and is influenced by, lipid metabolism—a therapeutic window for gliomas?

Metabolic reprogramming is a defining hallmark of cancer [109] and targeting metabolic pathways such as DNA synthesis with 5-FU is a foundational chemotherapeutic strategy. Targeting lipid metabolism has lagged, however, due to a lack of potency and side effects [110]. A renewed focus on targeting lipid metabolism centers on fatty acid synthase (FASN), a key enzyme of *de novo* lipogenesis that is often overexpressed in cancers associated with aberrant ErbB signaling [111]. FASN expression and EGFR signaling appear to be linked through a positive feedback loop in which RTK activation leads to increased FASN expression via sterol regulatory element-binding protein 1 (SREBP-1), and FASN overexpression activates EGFR [112,113]. Indeed, combining FASN inhibitors with the inhibitory EGFR antibody cetuximab results in strong synergistic anti-tumor effects in model cell lines [114,115]. It remains mechanistically unclear exactly how FASN overexpression alters EGFR signaling, but several groups have associated FASN overexpression with increased EGFR palmitoylation and altered cellular EGFR distribution—a trend explored further below [102,116]. A new FASN inhibitor, TVB-2640 (Denifanstat), was shown in a phase II trial to be well tolerated alongside anti-angiogenics for patients with recurrent glioblastoma multiforme (GBM), a particularly challenging cancer to treat [117].

The brain contains the second-highest lipid content across human tissues and tightly regulates lipid metabolism to maintain homeostasis [118]. Altered lipid metabolism is routinely associated with GBM [119,120]. Oncogenic signaling through EGFR gene amplification is observed in approximately 50% of GBM patients, often associated with the EGFRvIII mutation—a deletion of exons 2–7 that removes about half of the ECR, resulting in the inability to bind strongly to ligands and low-level constitutive kinase signaling [121,122]. Bi et al. recently reported that aberrant EGFRvIII signaling in GBM coincides with increased saturated PtdCho levels by amplifying lysophosphatidylcholine acyltransferase 1 (LPCAT1), an enzyme in the Lands cycle that remodels phospholipid fatty acid composition [123]. EGFR activation typically results in receptor internalization. However, Bi et al. found that increased PtdCho saturation leads to a preservation of EGFRvIII on the plasma membrane despite its constitutive activation. This finding suggests that changes in lipid metabolism may up-regulate EGFR signaling by preserving activated receptors at the PM. Therefore, promoting intracellular retention of EGFR away from the PM by pharmacologically altering lipid metabolism might be therapeutically beneficial. Indeed, off-target inhibition of sphingomyelin phosphodiesterase 1 (SMPD1) with the widely prescribed selective serotonin reuptake inhibitor (SSRI) fluoxetine shifts more EGFR to intracellular membranes and reduces aberrant EGFRvIII signaling in GBM [124]. SMPD1 is an acidic sphingomyelinase that converts SM to ceramide, and its inhibition results in the accumulation of many SM lipids [125]. Bi et al. showed that increased cellular concentrations of SM d18:1/n16:0 specifically reduce the amount of EGFRvIII at the plasma membrane, blunting EGFRvIII signaling [124]. The same authors also did a retrospective human study that revealed that patients taking fluoxetine concurrently with their GBM standard of care treatment had a ~75% increase in overall survival compared with patients taking standard of care alone or with other SSRIs. This work represents the first co-dependent lipid metabolism that appears to be pharmacologically actionable in EGFR-associated GBM. However, much more mechanistic work is needed to understand how intracellular retention of a signaling-competent EGFR mutant that has lost the ability to bind growth factors with high affinity could mitigate aberrant signaling. One possible explanation is that EGFRvIII is a substrate for WT EGFR and other ErbB receptors that provides novel pro-oncogenic signaling, as suggested by the EGFR-EGFRvIII-STAT signaling axis [126].

### Method advances enable the study of EGFR in native-like bilayers

Traditional approaches for studying RTK structure and function *in vitro* require solubilization of the membrane with detergents that replace the native lipid environment. Cryo-electron microscopy (cryo-EM) has recently been used to study multiple intact RTKs, including EGFR, but has so far failed to elucidate how the intracellular and extracellular domains are stereochemically linked across the bilayer [127-132]. The ECR is always well-defined in these studies, but the TM and ICRs have yet to be seen at atomic resolution in the context of the intact receptors, possibly due to a lack of anionic lipid–ICR interactions or because of destabilizing interactions with detergent micelles. New polymers have recently become commercially available that allow the extraction and purification of MPs alongside their native lipid compositions by creating ‘native nanodiscs’ [133,134]. Although these nanodiscs preserve MP–lipid interactions, they do not preserve important bilayer properties such as lipid packing density [135]. Native nanodiscs have been successfully used in many applications, including cryo-EM single-particle analysis, and are continuously being improved to overcome limitations such as divalent cation-induced instability [136-139]. Many groups are actively investigating native nanodisc usage in intact RTK studies, so we may soon know if native lipid compositions enable simultaneous observation of the ECR and ICR of EGFR and may also finally allow visualization of RTK interactions with lipid cofactors at atomic resolution.

Studies of higher-order EGFR signaling assemblies, such as receptor clusters, should ideally be carried out in continuous lipid environments like vesicles, since detergents and other small model membranes can constrain higher-order oligomerization. To this end, the Leahy lab has recently used extracellular vesicles and cryotomography to observe full-length EGFR ECR clustering at a resolution of ~15 angstroms [140]. This approach may pair well with EM methods that obtained high-resolution structures of the ion channel Slo1 from sonicated plasma membrane vesicles [141]. Cell-derived vesicles such as GPMVs could also provide a lens through which to understand how liquid–liquid lipid phase separation can influence MPs such as EGFR—a particularly vexing question. Probe-free ways to identify raft-like domains in GPMVs by cryo-EM have recently been published and may be insightful [142,143]. While currently outlandish, one foresees a day when all the techniques mentioned here could simultaneously provide insights into EGFR atomic structure, clustering, and local bilayer environment. Regardless, cell-derived and artificial vesicles will be crucial for understanding how lipids mechanistically influence EGFR function and opening the door to obvious next steps, such as looking into EGFR signaling complexes with downstream effector proteins.

## Conclusions

EGFR has been a defining model for RTK signaling in cancer, and the intense focus on its structure and function has largely overlooked the undeniable contribution of the membrane. Due to technical limitations, experimental studies of EGFR–lipid interactions have predominantly been observational, qualitative, and protein-centric. Hopefully, however, the work summarized in this review makes it clear that PM leaflet and lateral asymmetries provide unique lipid compositions that influence EGFR signaling through direct and indirect interactions. Pharmacologically targeting receptor–lipid interactions in cases of aberrant EGFR co-dependent lipid metabolism, such as in GBM, shows promise for much-needed therapeutic breakthroughs. Further, new technologies will enable a new era of RTK–lipid studies that mechanistically explain previous observations and may finally reveal how RTKs communicate across the bilayer to signal. Readers wanting a more in-depth review of EGFR–lipid interactions are directed towards [46].

**Perspectives**

- Epidermal growth factor receptor (EGFR) serves as a paradigm for receptor tyrosine kinase signaling and is an important therapeutic target in various cancer contexts; however, we still lack a clear understanding of how signaling is co-ordinated across (and influenced by) the membrane itself.
- EGFR signaling and cellular lipidomes influence each other; changes in lipid composition alter EGFR signaling, which in turn influences lipid remodeling pathways. The mechanistic details of how lipids directly and indirectly alter EGFR signaling remain largely unknown, although advances are being made.
- A continuous push to study membrane proteins in more *in vivo*-like environments and the potential for non-traditional therapies targeting lipid metabolism will reveal and exploit how complex bilayer environments influence EGFR signaling.

## Open Access

This article has been published open access under our Subscribe to Open programme, made possible through the support of our subscribing institutions, learn more here: https://portlandpress.com/pages/open_access_options_and_prices#conditional

## Abbreviations

C-tail: C-terminal tail
ECR: extracellular region
EGFR: epidermal growth factor receptor
FASN: fatty acid synthase
GBM: glioblastoma multiforme
GPMVs: giant plasma membrane vesicles
ICR: intracellular region
JM: juxtamembrane
LPCAT1: lysophosphatidylcholine acyltransferase 1
Ld: liquid-disordered
Lo: liquid-ordered
MP: membrane protein
PKC: protein kinase C
PM: plasma membrane
PtdCho: phosphatidylcholine
PtdEth: phosphatidylethanolamine
PtdIns: phosphatidylinositol
PtdIns(4,5)*P*_2_: phosphatidylinositol 4,5-bisphosphate
PtdSer: phosphatidylserine
RTK: receptor tyrosine kinases
SM: sphingomyelin
SMPD1: sphingomyelin phosphodiesterase 1
SSRI: selective serotonin reuptake inhibitor
TKD: tyrosine kinase domain
TKIs: tyrosine kinase inhibitors
TM: transmembrane
TrkA/B: tropomyosin receptor kinase A/B
cryo-EM: cryo-electron microscopy
